# Marine Structural Health Monitoring with Optical Fiber Sensors: A Review

**DOI:** 10.3390/s23041877

**Published:** 2023-02-07

**Authors:** Shimeng Chen, Jiahui Wang, Chao Zhang, Mengqi Li, Na Li, Haojun Wu, Yun Liu, Wei Peng, Yongxin Song

**Affiliations:** 1 Department of Marine Engineering, Dalian Maritime University, Dalian 116026, China; 2 School of Physics, Dalian University of Technology, Dalian 116024, China

**Keywords:** optical fiber sensor, structural health monitoring, marine structures, optical fiber applications

## Abstract

Real-time monitoring of large marine structures’ health, including drilling platforms, submarine pipelines, dams, and ship hulls, is greatly needed. Among the various kinds of monitoring methods, optical fiber sensors (OFS) have gained a lot of concerns and showed several distinct advantages, such as small size, high flexibility and durability, anti-electromagnetic interference, and high transmission rate. In this paper, three types of OFS used for marine structural health monitoring (SHM), including point sensing, quasi-distributed sensing, and distributed sensing, are reviewed. Emphases are given to the applicability of each type of the sensors by analyzing the operating principles and characteristics of the OFSs. The merits and demerits of different sensing schemes are discussed, as well as the challenges and future developments in OFSs for the marine SHM field.

## 1. Introduction

With the growing development of ocean resource exploitation, more and more marine infrastructure facilities and large structures, such as dams, bridges, submarine tunnels, oil and gas pipelines, and drilling platforms are being built. These facilities typically should have a lifespan of decades or even centuries. Due to the harsh marine environment, real-time structural health monitoring (SHM) and automatic data transmission are greatly needed towards eliminating potential hazards, improving the service life, and reducing maintenance costs. Hundreds and even thousands of sensors are usually needed to enable full-scale detection because of their huge sizes and super long spans (hm to km level) [1,2,3]. In addition, corrosion resistance, high pressure resistance, and extreme temperature variations are also the major issues to be considered for the monitoring systems in marine environments [4]. Traditionally, nondestructive SHM can be achieved by using the radioscope method [5], eddy current method [6], and Lamb wave method [7]. However, these methods require the use of expensive and large equipment. Recently, some other types of economical and light-based sensors, such as resistive strain gauges [8], piezoelectric elements [9,10], and optical fiber sensors (OFSs) [11,12,13,14,15,16,17], have been used for SHM.

As regards to the OFSs, they are advantageous on the aspects of small size, light weight, low power loss, anti-electromagnetic interference, corrosion resistance, extreme temperature resistance, and easy embedding [18,19,20,21] and thus show great potential for SHM in marine environments. This paper reviews the latest development and status of OFSs in the marine SHM field within the last ten years. It should be noted that while there are some review papers on optical fiber sensing, they are focused on marine environment detection, ocean observation, and ocean engineering [22,23,24]. For this review paper, emphases are given to the analysis, the characteristics, and applicable application scenarios in the marine SHM field of different types of OFSs. This review is organized into four sections. Following the introduction, Section 2 describes the classifications and principles of marine OFSs. Section 3 is narrowed down to the three most common typical marine OFSs (point sensing, quasi-distributed sensing, and distributed sensing). In this section, the working principles and latest applications of the different types of OFSs are summarized and reviewed in three sub-sections. In the Section 4, we give a conclusion and discuss a few market barriers associated with the OFSs’ application. Some future development proposals of OFSs in the marine SHM field are also put forward.

## 2. Classifications and Principles of Marine OFSs

Since the development of fiber optic technology by Charles Kao [25], fiber optic communication has developed at an amazing speed and has been widely applied. With the development of optoelectronic components, OFS has gradually attracted extensive attentions. The optical fiber is a kind of optical waveguide, which is made of silica or plastic with a large refractive index inside and a small refractive index outside that can be used as a light conduction tool. To provide mechanical protection for optical fibers, they are usually wrapped in a plastic coating. External perturbations will change the behavior of the light transmitted in the fiber core. For fiber optic communications, this external perturbation effect should be minimized. In contrast, the external induced effects are intentionally amplified for optical fiber sensing technology. The characteristic parameters of light (wavelength, intensity, phase, or polarization state) change as light travels through the fiber. As a variety of sensors such as temperature, pressure, displacement, strain, acceleration, and gyro, OFSs have been applied in many fields such as biomedicine [26], aerospace [27], the petrochemical industry [28], and so on.

Any marine sensor must be resistant to high pressure and extreme temperatures. Commercial optical fiber made of silica material can withstand pressures of approximately 2 × 10^5^ Psi and can withstand extreme temperatures ranging from −170 °C to 900 °C, approximately [29,30,31]. In addition, the single-ended OFS operation makes it ideal for conducting on-site marine inspections. Despite all these advantages, OFSs still have challenges in oceanography. Bio-fouling and corrosion must be considered for underwater sensors. In order to protect the optical fiber from environmental damage and interference, marine OFSs are usually packaged with materials such as steel and carbon-fiber-reinforced plastic [32].

**Figure 1:**
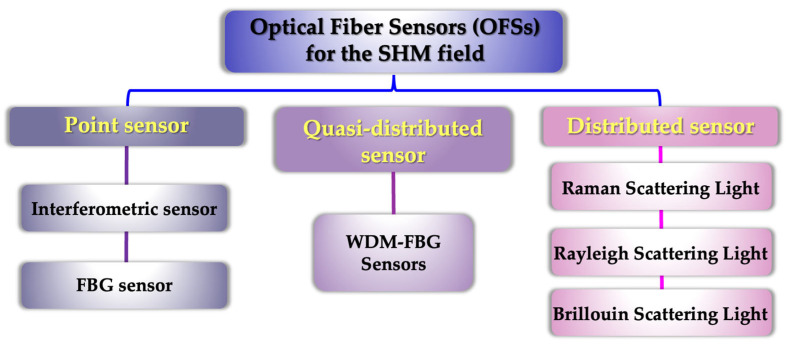
Overview of optical fiber sensing technologies for the SHM.

In the SHM field, OFSs are usually classified by the spatial distribution of the measured objects, as shown in Figure 1. OFSs can be classified as:

(i) Optical fiber point sensors, used for measuring the discrete points, that mainly include fiber Bragg grating (FBG) sensors [33,34] and interferometric sensors [35,36,37] in marine health monitoring. Since FBG sensors can present multiplexing capabilities for quasi-distributed sensors, its principle is described separately in Section 3.2. In Section 3.1, we mainly introduce the principle of interference sensors in detail.
(ii) The quasi-distributed sensor used for measuring is a set of regularly distributed spatial discrete points. As mentioned above, FBG is a point-like sensor with a small gauge length and can be used for single-point sensing. The FBG sensor has developed rapidly ever since the basic physics effect of FBG sensing was discovered. FBG based on the wavelength division multiplex (WDM) principle could realize the multiplexed arrays due to ultra-narrow spectral bandwidth. The quasi-distributed FBG sensing network connects multiple FBGs together using signal transmission fibers. It is one of the most popular wavelength-modulated sensors [38].
(iii) The distributed sensor is used to be continuously monitored in space. Different from point or quasi-distributed sensing, the distributed optical fiber sensors (DOFSs) can realize the detection of thousands of sensing points and offer the possibility of measuring variations along the entire optical fiber. DOFS can obtain test data in the spatial domain across a large distance by optical signal processing of backscattered light induced at any point located on the sensing fiber. DOFS mainly includes reflection, wavelength scanning, and interference methods. The reflection method is one of the most popular methods to measure the backscattering light in the process of optical fiber transmission, mainly including two different types: optical frequency domain reflectometry (OFDR) [39,40] and optical time domain reflectometry (OTDR) [41,42].

Among them, the optical fiber interferometer, FBG sensor, and DOFS are the most widely applied in the marine SHM field. The following sections focus on these OFSs in the SHM of marine infrastructure and geomorphology.

## 3. Typical Applications of OFSs for Marine SHM

### 3.1. Point Sensing (Interferometer)

The optical fiber interference sensor is one of the most sensitive fiber sensors, mainly including the Fabry–Perot interferometer (FPI) [43], Mach Zehnder interferometer (MZI) [44], Michelson interferometer [45], and Sagnac interferometers [46,47]. MZI and FPI are the most used in the marine SHM field.

#### 3.1.1. Working Principles

**Figure 2:**
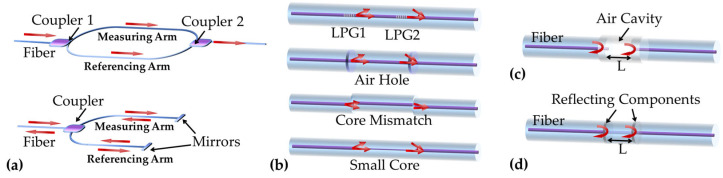
(**a**) Configurations of typical optical fiber MZI; (**b**) configurations of various types of in-line optical fiber MZIs. (**b**) Configuration of extrinsic (**c**) and intrinsic (**d**) FPI based on optical fiber.

MZI is an optical device used to detect the relative phase-shift changes between two collimating beams produced by splitting light from a source using the beam splitter. For typical optical fiber MZI, there are normally two independent fiber arms including the measuring arm and the reference arm [48,49,50], as shown in Figure 2a. Owing to the new technology of optical fiber micromachining, many in-line MZI configurations have been used [51,52,53,54,55]. The in-line optical fiber MZI could be based on a variety of micro-structured fiber sensing elements, as shown in Figure 2b [37,44,55]. This in-line approach allows the interferometer to be miniaturized and integrated. FPI is an interference cavity, and it is possible obtain the multiple superpositions of reflected and transmitted beams from the two reflectors. Common optical fiber FPIs are formed by making reflectors inside or outside of the fiber depending on the structure of the interferometric cavity [56,57]. They are usually classified into extrinsic FPIs (EFPI) or intrinsic FPIs (IFPI) [58,59], as shown in Figure 2c. In addition, the reflecting surface can also be the interfaces between two dielectrics or even Bragg gratings [60,61,62].

#### 3.1.2. Applications

**Figure 3:**
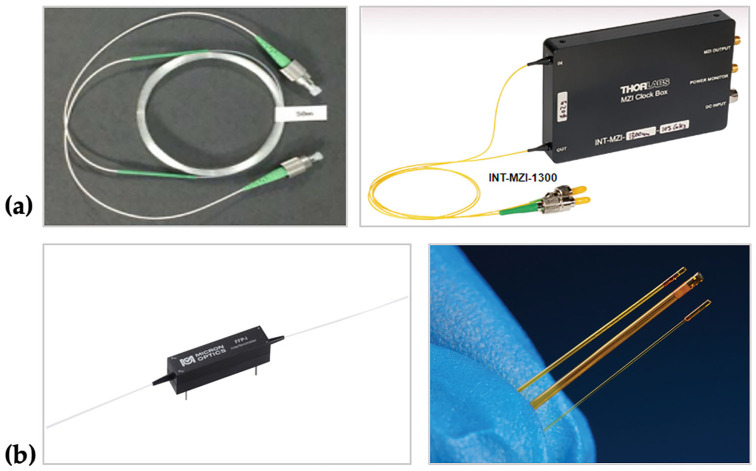
Several commercial optical fiber MZIs [12,13] (**a**) and FPIs [14,15] (**b**).

Optical fiber interferometers have been successfully used in applications by measuring changes of the optical cavity’s parameters. The optical cavity can be active (integrating a fiber laser sensor) [63,64,65] or passive (detecting the external parameters) [51,66,67,68,69,70]. As shown in Figure 3, commercial optical fiber MZI can be found from some companies, such as Optiphase Inc. (Berkeley, California, United States) and Thorlabs Inc. (Newton, New Jersey, United States) [71,72]. Many companies can offer commercial optical fiber FPI, such as Luna Inc. (Roanoke, Virginia, United States) and Fiso Inc. (Quebec, Canada) [73,74]. Many applications for measuring various types of parameters (strain, pressure, vibration, temperature, etc.) have been proposed using optical fiber interferometers due to their high sensitivity. However, this review paper is focused on marine SHM applications.

Through the detection of deep-sea pressure, underwater temperature, seabed sound waves, and so on, the optical fiber interferometer can realize the SHM of marine geomorphology (such as tsunamis and earthquakes) [75,76,77,78,79,80], submarine cables [81,82,83,84], offshore platforms [85,86,87], and other marine structures.

**Figure 4:**
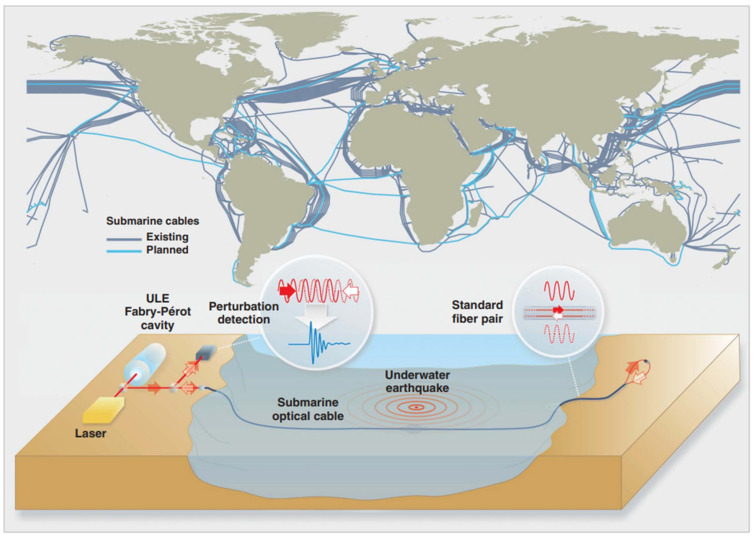
Detection of local and remote earthquakes using a laser based on FP cavities. Reprinted with permission from [75].

Marra et al. [75] produced a laser based on FP cavities, which is an ultralow expansion cavity. The light from the FP laser was injected at one end of the submarine link with an optical fiber pair. Different optical fibers correspond to different propagation directions, as shown in Figure 4. At the far end of the submarine link, two optical fibers are connected to form a loop so that the light returns to the transmitter. By measuring the phase difference of the returned optical signals and injected light source using a photodetector, the authors realized the detection of local and remote earthquakes. Furthermore, monitoring the seawater pressure also can detect tsunamis and earthquakes [88]. Qi et al. [76] proposed a small-size marine pressure measuring system including an ultra-high pressure optical fiber FP interferometer and miniaturized phase demodulating system. Pressure fatigue and hydrostatic pressure were tested in order to meet the requirements of marine pressure-testing applications. The experimental results showed that this sensor can steadily work in the range of 2–120 MPa for a long time. This sensing system can meet the requirements of pressure measurements throughout the ocean and can be applied to the ocean-profiling measurement program named the Argo plan. Duraibabu et al. [77] reported a novel miniature extrinsic FP interferometer for accurate measurement of marine pressure, which was mechanically robust, corrosion resistant, and suitable for underwater detection. This FP sensing system was mounted on a remotely operated underwater vehicle (ROV) to detect the pressure variation by online monitoring the reflected optical spectrum. The operating performances of this sensor exceed those of commercial ROV-mounted sensors, such as accuracy (25 mm) and resolution (5 mm). In addition, fiber optic hydrophones based on the interference principle can be used for SHM through acoustic sensing. It is very suitable for SHM with the slot type damage, such as underwater earthquake and pipeline leakage [89,90,91]. For example, Jin et al. [79] discussed and validated a fiber optic vector hydrophone based on FP interferometry. This vector hydrophone combined the advantages of small size, low cost, and high reliability.

**Figure 5:**
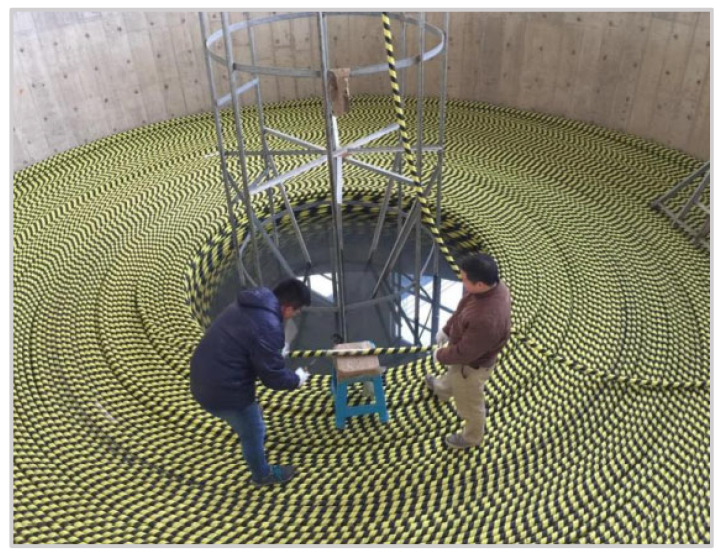
Online submarine cable monitoring system using bidirectional MZ interferometer. Reprinted with permission from [81].

With the continuous development of global communications, gigameters of submarine cables encircle the global seabed. As an important infrastructure, it is very important to monitor the damage and temperature of submarine cable. At present, the submarine cables are typically submerged to a depth of several kilometers in the deep sea [92,93]. The submarine optical fiber composite cable unit has been developed rapidly because it can realize cable monitoring while transmitting power without increasing the cost [94]. Gao et al. [81] designed an online monitoring system based on a bidirectional MZ interferometer for submarine cable. Different optical fibers inside the submarine cable were selected as the sensing arms of the MZ interferometer. During the vibration positioning tests, the submarine cable was placed in the cable pool, as shown in Figure 5. The authors knocked on different locations of submarine cable at different depths and real-time monitored the sensing response signals. The experimental results showed that this MZ interferometric sensor system can effectively monitor the vibration events of submarine cables, and the average positioning error was 13.23 m. Wang et al. [84] reported a double MZ distributed optical fiber sensing technology for monitoring submarine cables. The MZ vibration sensing system is designed both in software and hardware. An optimized measuring scheme was put forward in anticipation of the possible problem of false alarm in the future application of the monitoring system.

For the past few decades, the OFS application in the gas and oil industry has grown substantially. It has been used for monitoring offshore platforms (such as pipelines and downholes) by detecting the temperature, the pressure, and so on. Among them, optical fiber interferometers have been widely applied for the detection of pipeline leakage and downhole pressure [85,86,87]. However, they have become commercialized on drilling platforms without revolutionary technological innovations in the past ten years.

Due to the local single-point sensing characteristics, optical fiber FP interferometers are mainly focused on submarine earthquakes, and optical fiber MZ interferometers are mainly focused on submarine cables. The OFSs based on interferometers and corresponding main marine monitoring contents are presented in Table 1.

### 3.2. Quasi-Distributed Sensing (WDM-FBG)

Quasi-distributed sensing technology can realize multi-point simultaneous detection. In the optical fiber sensing field, the quasi-distributed sensing usually refers to WDM-FBG technology.

#### 3.2.1. Working Principle

**Figure 6:**
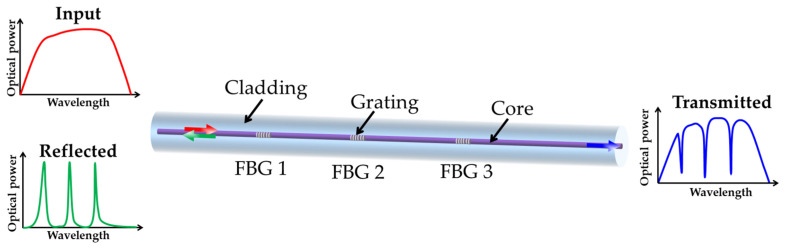
Operating principle of quasi-distributed FBG sensor.

FBG is formed by inducing a periodic RI perturbation along the length of the fiber core [95,96]. As a selective optical filter, FBG could reflect a part of the incident with the selected wavelength while the rest of the incident light passes through. The Bragg wavelength is related to the grating period, which is altered by tension or compression (such as mechanical or thermal loads). In general, the quasi-distributed optical fiber sensing system is actually a multiplexing system of multiple discrete OFSs, including WDM, time division multiplexing, frequency division multiplexing, and space division multiplexing. The WDM-FBG sensing system can be realized by writing several FBGs with different periods and/or effective RI in the same fiber. Figure 6 shows that the WDM-FBG sensor can clearly distinguish different Bragg wavelengths using the same optical fiber line. The in-line optical connection property of FBG makes it feasible to build up fiber optic sensing networks [97].

#### 3.2.2. Applications

**Figure 7:**
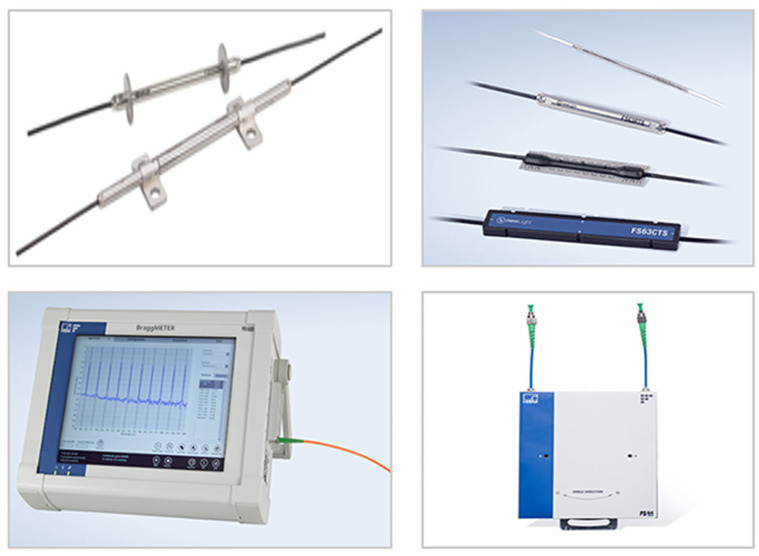
Several commercial FBG sensing elements and units [103,104].

Unlike the local point sensors, quasi-distributed sensors are suitable for monitoring large structures because there is no need to install transmission fibers at each test site separately [98]. Due to its reliability and robustness, the WDM-FBG sensor has revealed great application potential at quasi-distributed sensing fields of temperature, strain, pressure, and ultrasound detection [99,100,101,102]. As shown in Figure 7, FBG is one of the most mature OFSs at present, and many companies sell photoelectric products or transducers based on FBG sensing technology, such as Roctest Inc. (Saint-Lambert, Canada) and HBM Fibersensing Inc. (Darmsdart, Germany) [103,104]. At present, the quasi-distributed FBG sensors have already been applied in a wide range of industries. SHM is the most active area of application for quasi-distributed FBG sensors [105,106,107,108,109,110,111,112,113]. Several FBG sensing elements could be embedded or attached to the monitoring structures and connected to an optical fiber sensing network. At present, more than half of the SHM-OFS projects have opted for quasi-distributed FBG sensors [114]. This paper focuses on the marine SHM field. FBG sensors have demonstrated superior performance in the long-term real-time health monitoring of marine areas.

Due to its characteristics of multi-point monitoring, a quasi-distributed FBG sensor can realize the health monitoring of marine structures such as drilling platforms [112,115,116,117,118,119,120,121,122,123,124], submarine pipelines [116,125,126,127], bridges [3,128,129,130,131,132,133], dams [134,135,136,137], and hulls [15,138,139,140,141,142,143,144,145].

Compared with the traditional sensors, FBG sensors offer the possibility of strain and temperature measurements under some harsh conditions, for example, of 20–200 °C temperature [146,147] and 0.1–100 MPa pressure [148,149]. Therefore, FBG sensors with good stability and large operating range can be used for long-term downhole monitoring. Xu et al. [115] developed an FBG-based bundle-structure riser stress-monitoring sensor to meet the requirements of riser safety monitoring in offshore oil fields. A 49-day marine test in water depths of 1365 m and 1252 m was carried out on the “HYSY-981” ocean oil drilling platform. This sensor was installed on the risers without welding and pasting, making the installation convenient, reliable, and harmless to the risers. The testing results agreed basically with the mechanical simulation results. Wang et al. [119] explored different FBG packaging materials applied in the offshore drilling platform in the salt-fog environment. Authors chose corrosion-resistant packaging materials (FR-4 epoxy board, sheet molding compound, and sheet molding compound) for the FBG sensing element and realized the improvement of corrosion resistance and sensitivity of the sensing system. This work offered useful information for OFS development in the marine SHM field. The dynamic response of the submarine oil pipeline under external force or seismic excitation is a coupled vibration of liquid and solid interaction. Due to its advantages of being explosion proof and having high accuracy, the FBG sensor is suitable for monitoring the response caused submarine pipeline leakage. Cabral et al. [127] demonstrated an approach to monitoring a pipeline’s bonded joints during assembly and operation using FBG sensors embedded into the joints’ adhesive layer. This approach was shown to be adequate to monitor the assembly of the joints and the pipelines, effectively covering all stages of the pipeline’s lifecycle. This work can find wide use for monitoring plastic and composite pipelines that make use of adhesive-bonded joints. Zhou et al. [125] experimentally studied the dynamic characteristics of FBG sensors and commercial strain gauges fixed to the underwater pipeline. The theoretical and experimental results showed that the FBG sensor was superior to a commercial strain gauge and satisfied the dynamic monitoring requirements of submarine pipeline.

FBG sensors have shown good performance for marine SHM of civil engineering composite structures, ensuring their structural reliability, durability, and integrity. The real-time SHM of long-span bridges is one of the most representative applications for FBG sensors [150,151]. Yan et al. [3] designed the SHM system for the Hong Kong-Zhuhai-Macao Bridge. In total, 277 sensors were installed on the section of the Qingzhou Shipping Channel Bridge, with the largest including a lot of FBG temperature sensors and FBG strain sensors in different locations. For this monitoring system, the FBG sensors possessed good time-frequency resolution compared with other types of sensors. Hu et al. [131] developed an FBG vibration sensor for online monitoring of the cable vibration characteristics of Tongwamen Bridge. The monitored vibration frequency was converted into cable force according to the string vibration theory. The FBG arrays were mounted symmetrically on 8 of 19 cables to achieve an indirect measurement of bridge cable force. In addition, an FBG liquid-level system as the SHM-OFS has been used in large infrastructures [152]. For example, Rodrigues et al. [128] applied FBG liquid-level sensors to concrete bridges. This methodology is based on a hydrostatic leveling system and the application of the communicating vessels principle to an internal hydraulic system, which is installed along the structure and reaches the relevant points wherein the relative vertical displacement is going to be measured. This sensing system with a total of 30 optical-based strain transducers was successfully applied for the Lezíria Bridge.

**Figure 8:**
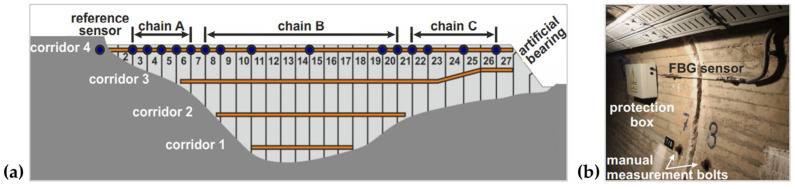
SHM within the hydro power dam using quasi-distributed FBG sensor. (**a**) Layout scheme; (**b**) one of the installed FBG sensors within the maintenance corridors of the dam. Reprinted with permission from [134].

Similar to bridges, dams are also the common application area for FBG sensors due to their enormous size. In recent years, many hydro power plants were operated by pumped storage, which requires additional equipment available for monitoring. An FBG-based monitoring system was reported by Monsberger et al. [134] and successfully installed inside a hydro power dam. This FBG sensing system possessed a very high spatial resolution (millimeter level) by using an optical backscatter reflectometer. As shown in Figure 8a, there were 15 concrete joints with FBG sensing elements in one of the maintenance corridors, and the whole measuring chain was divided into three separate chains. The FBG sensing unit for each link was mounted above the manual measurement bolt and can be measured individually (Figure 8b). The experimental results demonstrate that the optical backscatter reflectance method is suitable for analyzing FBG networks. Regina et al. [137] designed an FBG-based inclinometer for landslide monitoring in dams. By detecting lateral displacements, the cubic spline interpolation method was used to reconstruct the tube profile. The testing results showed a good agreement between the curve reconstruction and the plotted data of field measurements.

**Figure 9:**
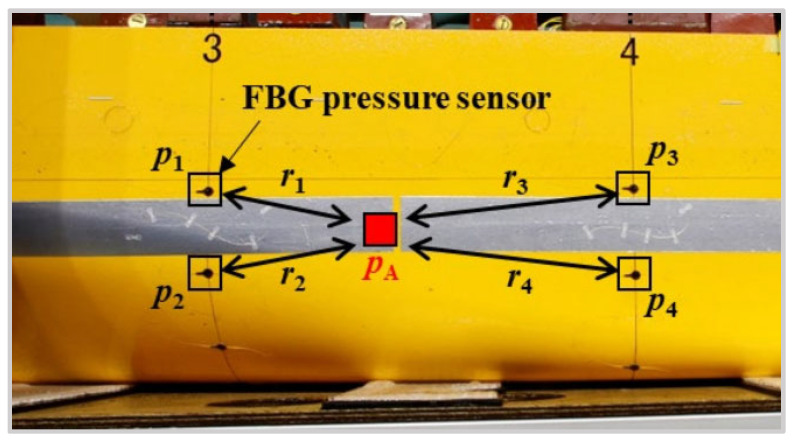
Hull structural strength evaluation using FBG pressure sensors. Reprinted with permission from [138].

The military, such as the United States Navy, has shown great interest in the OFS application for ships [153,154]. Among the OFSs, the FBG sensor has received a lot of attention because it can be used for SHM in composite-hulled crafts. Komoriyama et al. [138] used FBG pressure sensors for hull structural strength evaluation. The towing tank test was carried out with an elastic ship model to investigate the FBG reliability for strength evaluation. By installing FBG sensors outside and inside the hull, the authors obtained actual water pressure. Furthermore, the vertical bending moment was obtained by interpolation algorithm and finite element analysis. The interpolation algorithm for pressure on the hull’s surface illustrated that point A was interpolated by using that of points 1 to 4 in Figure 9. The test results verified that the water pressure measurement based on the FBG sensor was effective in evaluating the strength of hull structural strength. Temperature monitoring is very important to evaluate the thermodynamic performance of the auxiliary machinery, piping, and chillers of traditional ships or hydrogen and natural gas storage tanks of new energy ships. The FBG sensor has promoted the development of the hull safety monitoring system for the past few years. Han et al. [139] employed FBG sensing technology to monitor the temperature of a cryogenic storage tank, pipeline, and water chiller. Through a series of experiments in a wide temperature range, the FBG sensors with temperature-sensitive metal coating materials were proved to have better reliability for long-term temperature measurements and higher safety than those of the traditional thermistors. In addition, other interference parameters, such as humidity and vibration, had little impact on the temperature response of FBG. This work provided supports and references for the safety performance test platform of the ship.

Considering quasi-distributed sensing characteristics, the above examples demonstrated that FBG sensors are particularly suitable for the large-size marine SHM of offshore platforms, bridges, dams, hulls, etc. OFSs based on the WDM-FBG and corresponding main marine monitoring contents are presented in Table 2.

### 3.3. Distributed Sensing (DOFS)

For distributed sensing systems, scattered signals can be used for monitoring along the entire length of the fiber. DOFS serves as both a transmission fiber and transducer in the sensing system, which is one of the best potential applications of optical fiber sensing technology.

#### 3.3.1. Working Principle

**Figure 10:**
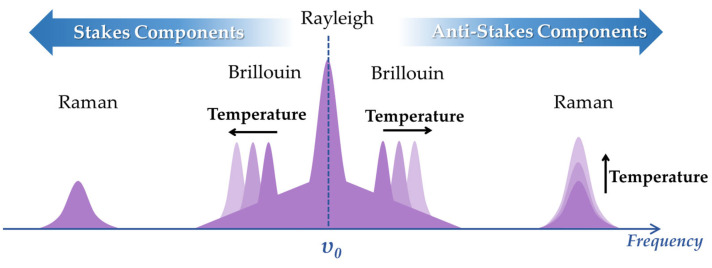
Light emission from Rayleigh, Raman, and Brillouin scattering.

When a ray, which could be of any wavelength, is emitted into an optical fiber, most of the light travels through the fiber, while a small fraction is backscattered. The property information of the optical fiber affected by the environment can be provided by the backscattered light. There are three different basic scattering theories for DOFS: linear Rayleigh scattering [155], nonlinear Raman scattering [156], or Brillouin scattering [157]. The scattered light can be categorized into the three wave bands, and the schematic spectra are shown in Figure 10. Rayleigh scattered light possesses the same wavelength as the light source, whereas wavelengths of scattered light shift for Brillouin and Raman. Rayleigh scattering in DOFS technology is primarily employed to test propagation effects (such as attenuation and gain, polarization variation, or phase interference). So, Rayleigh scattered light in optical fiber is sensitive to fiber deformation and variation of temperature or magnetic field. DOFS-based Rayleigh scattering is widely applied in strain [158] and temperature [159] measurement. Raman scattering induces a frequency shift interrelated with the stretching modes between atoms, which depends on the temperature variation [160]. The temperature dependencies of Stokes and anti-Stokes Raman scattering are described in Figure 10 [161]. This makes DOFS sensitive to temperature change. DOFS based on Raman scattering is usually applied in distributed temperature sensing (DTS) using the OTDR or OFDR method [162,163,164,165]. Brillouin scattering is intrinsically dependent on the fiber density, which in turn depends on temperature and strain, since Brillouin scattering can be applied for temperature and strain DOFSs [166,167].

#### 3.3.2. Applications

**Figure 11:**
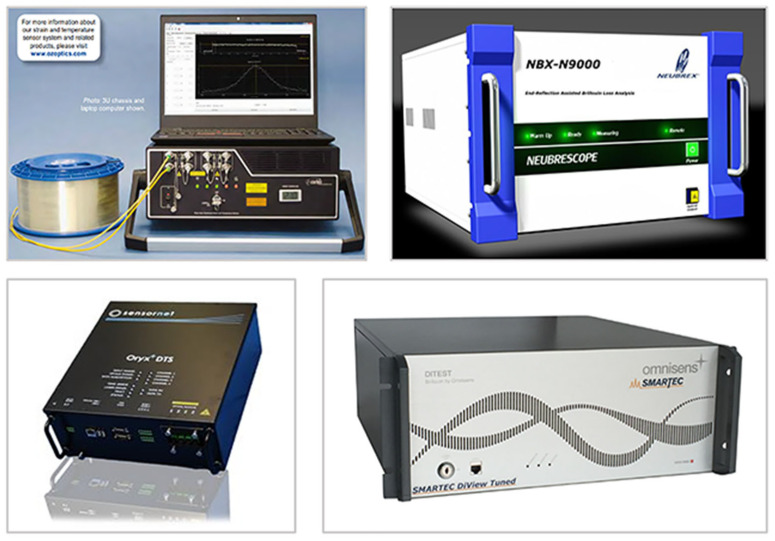
Several commercial DOFS optoelectronic devices [168,169,170,171].

In addition to the advantages of optical fiber sensing, an additional benefit associated with DOFS is that it requires only a single connected optical cable to communicate data, in contrast to the large number of optical cables required for discrete OFSs. DOFS serves as a unique single-ended monitoring technique that uses the backscattered light of the fiber to feed back the performance of the fiber. It can provide a global behavior of the large-scale structure, rather than extrapolation from a finite number of measurement points. Several companies have realized the commercialization of DOFS, such as Sensornet Inc. (Watford, United Kingdom), Neubrex Inc. (Kobe, Japan), OZ Optics Inc. (Ottawa, ON, Canada), and Smartec Inc. (Manno, Switzerland), as shown in Figure 11 [168,169,170,171]. DOFS has been widely applied in the measurement of temperature, strain, and vibration, especially in the field of marine structural health monitoring.

Because DOFSs can realize remote and continuous sensing, they can most commonly realize the SHM of underwater cables [172,173,174,175,176,177,178,179], oil and gas pipelines [176,180,181,182,183,184], and dams and tunnels [185,186,187,188,189].

**Figure 12:**
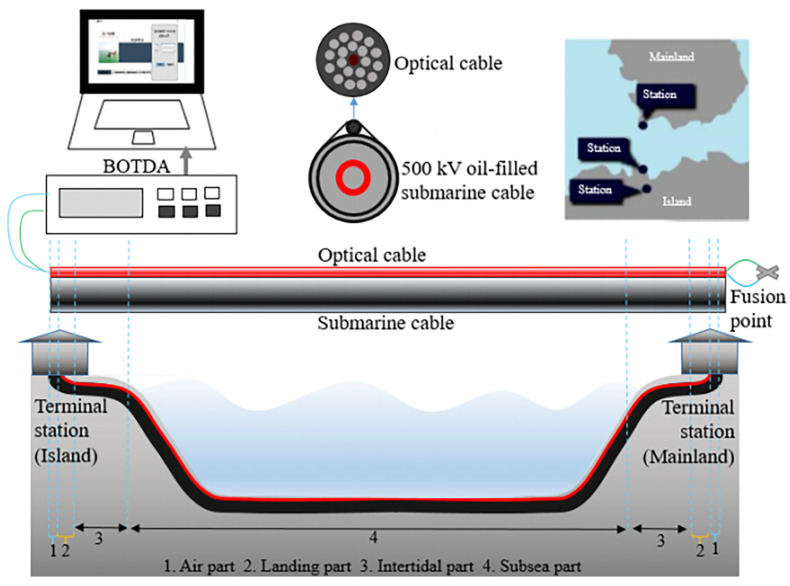
Submarine cable temperature monitoring in China’s southern coast using BOTDA sensing system. Reprinted with permission from [172].

Recent developments of DOFS allow the monitoring of up to 300 km by using optical amplifiers. This means DOFSs are well suited for detecting long-distance submarine cables. Chen et al. [172,173] established a Brillouin optical time domain analysis (BOTDA) distributed optical fiber monitoring system for monitoring the temperature of high-pressure oil-filled submarine cables by bundling the optical cables and power cables together. The sensing system setup and installation method are shown in Figure 12. The special sealing and joint structures were designed to meet the accuracy calculation method and monitoring system based on the onshore simulation platform. In addition, the optimized sensing system was used on the 500 kV submarine cable of the Hainan networking system to monitor sudden temperature changes caused by instantaneous overload and external losses. Huang et al. [174] established the all-fiber BOTDA monitoring system to monitor the surface temperature of submarine cables. Authors measured the conductor current by using an optical fiber current transducer and calculated the conductor temperature of the submarine cable. Compared to traditional current sensors, the optical fiber current transducer only monitored the cable conductor current, which can eliminate the effects caused by long-distance overhead lines and compensating reactor. This is a good way to achieve performance optimization that was used in the Hainan interconnection project. For monitoring shock events of submarine cables, Fouda et al. [175] used phase-sensitive OTDR to detect vibration signals from the optical fiber on cables. The vibrational pattern recognition of optical fibers was implemented by using time-frequency domain features and a support vector machine to determine the magnitude of the event. A lot of experimental data showed that this method can effectively identify the disturbance events of submarine cables. DOFS applications in temperature and vibration monitoring of submarine cables are important for the reliability of submarine cable operation.

Similarly, the application of DOFS to long-distance pipelines in the oil and gas industry is of great interest and has therefore seen a substantial increase. Feo et al. [180] presented pioneering investigation in the DOFS application for monitoring risers. This team conducted well-level experiments by simulating an offshore riser environment. The downhole distributed sensor involving optical fiber DTS and distributed acoustic sensing (DAS) was instrumented on the experimental setup by using metallic clamps. For flexible riser monitoring, DOFS could be installed on one of the metal rods to form an umbilical. DOFS can provide real-time and accurate monitoring data for the sake of effective well control. In order to implement subsea pipeline (1.3 km long) installation inspection, Cementys company [181] designed a SensoluxTM sensor based on Raman and Brillouin OTDR. This sensor cable contains four optical fibers to measure the Raman scatterings (temperature) and the Brillouin scatterings (strain and temperature). For protecting the optical cables, they were glued into grooves in the concrete surrounding the metallic pipe. By measuring the strain conditions of the pipeline during different steps (such as lay or tow), the pipeline could be certificated. In addition, Inaudi and Glisic [182] proposed a successful application of DOFS monitoring of a gas pipeline near Rimini, Italy. DOFS could measure thousands of points along a single fiber and possesses unique features compared with traditional technology. It is great for monitoring oil and gas pipelines and optimizing oil production.

**Figure 13:**
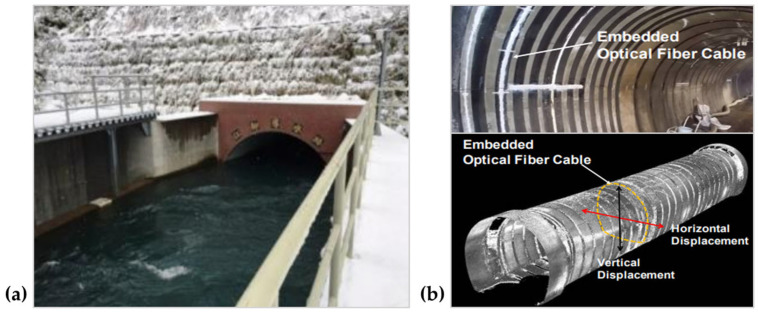
Real-time strain monitoring of the aqueduct tunnel using DOFS. (**a**) The end of the subject aqueduct tunnel. (**b**) Embedded optical fiber cable in the retrofitted tunnel. Reprinted with permission from [185].

SHM systems based on DOFS are also very valuable in dams and subsea tunnels. Imai et al. [185] installed the Brillouin optical correlation domain analysis (BOCDA) around the interior circumference of the aqueduct tunnel and real-time monitored strain distribution. Figure 13 shows the aqueduct tunnel of a hydropower plant and the DOFS installed in the retrofitted tunnel. The fiber cable was attached in the trench of concrete lining by use of epoxy adhesive. The fiber cable was wired out to the end of the tunnel and connected to an optical analyzer. By calculating the cross-section deformation, continuous monitoring of tunnel convergence could be realized. This method avoided power outages and drainage operations. Similarly, Wang et al. [186] monitored Nanjing Yangtze Shield Tunnel for 55 days using optimized DOFS. The sensing elements installed on the 90-m-long tunnel ring successfully monitored temperature and strain. Pumped-storage power stations are subject to external forces and environmental erosion. It is necessary to perform long-term SHM for avoiding economic loss and safety hazards. Liang et al. [187] installed the DOFS on the dam construction site of the Liaoning Qingyuan pumped storage station to monitor the temperature during the concrete curing process. The monitoring results successfully revealed the temperature variation of the concrete curing process.

Based on the intrinsic characteristics of DOFS, it is well-suited to detect the determinants with large spatial size and large span, especially the submarine cables, pipelines, and tunnels. OFSs based on the DOFSs and corresponding main marine monitoring contents are presented in Table 3.

## 4. Conclusions and Outlooks

Three types of typical OFSs (optical fiber interferometers, WDM-FBG sensors, and DOFSs) for marine SHM are discussed in this paper. Compared to other marine SHM methods, OFSs show superior performances in monitoring structural strain, stress, vibration, temperature, displacement, etc. It should be noted that the applications of OFSs in the marine field are still under-developed and have some challenges and great potential, from both theoretical and engineering aspects.

(a) Novel optical fiber sensing structures and new smart materials are greatly needed for continually improving the detection sensitivity. They are the main avenues of designing new optical fiber sensing structures or fabricating optical fibers using new materials or technologies to be increased. Furthermore, combining machine-learning algorithms to improve the performance of optical fiber sensing systems is a major current approach.
(b) Artificial intelligence should be paid more and more attention to for solving the cross-talking problems, such as solving the multi-parameter cross-sensitivity by combining artificial intelligence and machine learning. Traditionally, these problems were solved by using additional sensing elements to measure the interference parameter. Using artificial intelligence, the effective signal could be separated from the mixed optical signals more cheaply and efficiently.
(c) Development on the installation techniques is greatly desired. The installation of OFSs for deep-sea marine structures is very difficult due to the inapproachable deep-sea environment for human beings. For optical fiber point sensors, the combination of OFSs and ROV for measurement will be the trend in the marine SHM field; for quasi-distributed and distributed fiber sensors, seismo-acoustic sensors using existing fiber optic seafloor telecom cables have great potential. Combining FOSs with existing submarine cables is a growing trend.
(d) There are also many perturbations in the harsh ocean environment, especially the external damage caused from different sources. How to protect the fragile fibers from damage while bettering transfer deformation, vibration, and other information requires further improvements in fiber packaging technology.

It is believed that with the continued development of the optical fiber sensing technologies, OFSs are expected to play more and more important roles in marine SHM in the near future.

## Figures and Tables

**Table 1:** Interferometric OFSs for SHM.

Monitoring Item	Sensors and Configuration	Variables	Authors	Year
Submarine earthquake	FP laser based on ultralow expansion cavity	Phase difference	Marra et al. [75]	2018
	Miniaturized FP pressure measuring system	Pressure	Qi et al. [76]	2019
	Fiber vector hydrophone based on FP interferometry	Acoustic	Jin et al. [79]	2018
Damage of submarine cable	Bidirectional MZ interferometer	Vibration	Gao et al. [81]	2020
	Double MZ distributed optical fiber sensing system	Vibration	Wang et al. [84]	2014

**Table 2:** WDM-FBG-based OFSs for SHM.

Monitoring Item	Sensors and Configuration	Variables	Authors	Year
Drilling platforms	FBG-based bundle-structure riser stress monitoring sensor	Stress	Xu et al. [115]	2015
	FBG sensors embedded into the joints’ adhesive layer	Strain	Cabral et al. [127]	2020
Bridges	FBG-based temperature and strain sensing arrays	Temperature/ Strain	Yan et al. [3]	2019
	FBG arrays based the theory of string vibration	Vibration	Hu et al. [131]	2017
Dams	FBG monitoring system using an optical backscatter reflectometer	Strain	Monsberger et al. [134]	2017
	FBG-based inclinometer arrays fixed along a flexible tube	Displacement	Regina et al. [137]	2021
Hulls	FBG sensors based on finite element analysis	Pressure/ Strain	Komoriyama et al. [138]	2020
	FBG sensors with temperature-sensitive metal coating materials	Temperature	Han et al. [139]	2021

**Table 3:** DOFSs for SHM.

Monitoring Item	Sensors and Configuration	Variables	Authors	Year
Submarine cables	BOTDA distributed optical fiber monitoring system	Temperature	Chen et al. [172,173]	2022
	All-fiber BOTDA monitoring system	Temperature	Huang et al. [174]	2019
	Phase-sensitive OTDR to detect vibration	Vibration	Fouda et al. [175]	2021
Oil and gas pipelines	DOFSs involving DTS and DAS	Temperature/ Acoustic	Feo et al. [180]	2020
	SensoluxTM sensor based on Raman and Brillouin OTDR	Strain/ Temperature	Cementys company [181]	2017
Tunnels	BOCDA-based optical fiber strain sensor	Strain	Imai et al. [185]	2021
	DOFSs based on Brillouin frequency shift	Displacement	Wang et al. [186]	2019

## Data Availability

Not applicable.
